# Inhibiting AKT Phosphorylation Employing Non-Cytotoxic Anthraquinones Ameliorates T_H_2 Mediated Allergic Airways Disease and Rhinovirus Exacerbation 

**DOI:** 10.1371/journal.pone.0079565

**Published:** 2013-11-05

**Authors:** Caio Cesar de Souza Alves, Adam Collison, Luke Hatchwell, Maximilian Plank, Matthew Morten, Paul S. Foster, Sebastian L. Johnston, Cristiane França da Costa, Mauro Vieira de Almeida, Henrique Couto Teixeira, Ana Paula Ferreira, Joerg Mattes

**Affiliations:** 1 Department of Parasitology, Microbiology and Immunology, Institute of Biological Sciences, Federal University of Juiz de Fora, Minas Gerais, Brazil; 2 Experimental&Translational Respiratory Group, University of Newcastle and Hunter Medical Research Institute, Newcastle, Australia; 3 Priority Research Centre for Asthma and Respiratory Diseases, University of Newcastle and Hunter Medical Research Institute, Newcastle, Australia; 4 Airway Disease Infection Section, National Heart and Lung Institute, Medical Research Council & Asthma UK Centre in Allergic Mechanisms of Asthma, Imperial College London, London, United Kingdom; 5 Department of Chemistry, Federal University of Juiz de Fora, Minas Gerais, Brazil; 6 Paediatric Respiratory & Sleep Medicine Unit, Newcastle Children’s Hospital, Newcastle, Australia; Institute for Virus Research, Laboratory of Infection and Prevention, Japan

## Abstract

**Background:**

Severe asthma is associated with T helper (T_H_) 2 and 17 cell activation, airway neutrophilia and phosphoinositide-3-kinase (PI3K) activation. Asthma exacerbations are commonly caused by rhinovirus (RV) and also associated with PI3K-driven inflammation. Anthraquinone derivatives have been shown to reduce PI3K-mediated AKT phosphorylation *in-vitro*.

**Objective:**

To determine the anti-inflammatory potential of anthraquinones *in-vivo.*

**Methods:**

BALB/c mice were sensitized and challenged with crude house dust mite extract to induce allergic airways disease and treated with mitoxantrone and a novel non-cytotoxic anthraquinone derivative. Allergic mice were also infected with RV1B to induce an exacerbation.

**Results:**

Anthraquinone treatment reduced AKT phosphorylation, hypoxia-inducible factor-1α and vascular endothelial growth factor expression, and ameliorated allergen- and RV-induced airways hyprereactivity, neutrophilic and eosinophilic inflammation, cytokine/chemokine expression, mucus hypersecretion, and expression of T_H_2 proteins in the airways. Anthraquinones also boosted type 1 interferon responses and limited RV replication in the lung.

**Conclusion:**

Non-cytotoxic anthraquinone derivatives may be of therapeutic benefit for the treatment of severe and RV-induced asthma by blocking pro-inflammatory pathways regulated by PI3K/AKT.

## Introduction

It is estimated that asthma affects more than 300 million people worldwide [1]. Asthma is characterized by airways hyperreactivity (AHR) and inflammation, mucus hypersecretion, and an aberrant T cell response [2]. Accumulation of eosinophils, neutrophils and lymphocytes in the airways correlate with disease severity and anti-inflammatory treatments ameliorate episodic airways obstruction, which is the clinical hallmark of asthma [3,4]. Airways inflammation is tightly regulated and involves the release of interleukin (IL)-13 and IL-5 produced by T helper type 2 (T_H_2) cells and natural killer T cells, IL-17 by T_H_17 cells, as well as chemokines by residential lung and innate immune cells [5–7]. IL-13 also induces mucus production in a signal transducer and activator of transcription 6 (STAT6)-dependent manner and is both sufficient and required for the development of AHR and goblet cell hyperplasia [8–10]. Acute asthma exacerbations are a major disease burden and predominantly caused by infection with rhinovirus (RV) -the common cold virus- [11–14]. Importantly some asthmatics display impaired innate immune responses with deficient type 1 and 3 interferon (IFN) responses upon RV infection [15,16]. Corticosteroids are the mainstream anti-inflammatory treatment used in asthmatics and have been effective in reducing eosinophilic inflammation. However, their therapeutic efficacy in preventing neutrophilic and RV-induced inflammation and symptoms is thought to be limited [17]. Furthermore, side effects can be observed in asthmatics requiring high dose or systemic corticosteroid treatment. In some patients asthma is steroid-resistant and then difficult to control [18,19]. Thus novel therapeutic strategies for severe asthma and RV-induced exacerbations are required.

Mitoxantrone belongs to the clinically useful topoisomerase 2-targeting anthraquinones that is used to treat metastatic breast cancer, leukemia, and lymphoma [20–22]. Mitoxantrone disrupts DNA synthesis and repair causing single and double-strand breaks by intercalating the DNA through hydrogen binding [23] resulting in non-specific cytotoxicity [20–22]. In patients with relapsing multiple sclerosis mitoxantrone may be of therapeutic benefit as a potent immunosuppressive agent targeting proliferating macrophages, B and T lymphocytes [24,25]. Recently topoisomerase 2-independent effects of mitoxantrone have also been described that may be of relevance for therapeutically modulating the aberrant immune response observed in asthma and RV-induced exacerbations. Specifically, mitoxantrone inhibited hypoxia-inducible factor (HIF)-1α and vascular endothelial growth factor (VEGF) expression through dephosphorylation of AKT in transformed cell lines suggesting that mitoxantrone regulates the phosphoinositide-3-kinase (PI3K)/AKT pathway [26]. 

Importantly several lines of evidence suggest that inhibition of the PI3K effector pathway may be a potential therapeutic strategy for asthma and RV-induced exacerbations [27–29]. Recently it has been shown that the T_H_2 cytokine IL-25 promotes angiogenesis, at least in part, by increasing VEGF/VEGF receptor expression through PI3K/and Erk/MAPK pathways [30]. Osteopontin is upregulated by epithelial cells and macrophages in the lungs of asthmatics that in turn activates PI3K/AKT downstream signaling pathways to induce IL-13, AHR, mucus hypersecretion, and pro-matrix metalloproteinase-9 in the lungs [31]. Receptor-mediated mast cell growth, differentiation, homing to their target tissues, survival and activation are also controlled, to varying degrees, by PI3K-driven pathways [32]. Finally, PI3K/AKT signaling is required for maximal RV-induced neutrophilic airway inflammation in an experimental mouse model and RV-induced IL-8 expression by airway epithelial cells, likely via its essential role in virus internalization [33,34]. 

In this study, the effects of mitoxantrone and a novel non-cytotoxic anthraquinone derivative (*O,O´-didodecanoyl-1,4-dihydroxyanthraquinone*) on allergic airways disease (AAD) and RV-induced exacerbation was investigated. We show that anthraquinone treatment reduced AKT phosphorylation, HIF-α and VEGF expression, and ameliorated allergen- and RV-induced airways hyprereactivity, neutrophilic and eosinophilic inflammation, cytokine/chemokine expression, mucus hypersecretion, and expression of T_H_2 factors in the airways. Anthraquinones also boosted type 1 interferon (IFN) responses and limited RV replication in the lung. 

## Methods

### Animals

Male BALB/c mice, 6-8 weeks old, were obtained from the Specific Pathogen Free Facility of the University of Newcastle. The Animal Care and Ethics Committee of the University of Newcastle, Australia approved all experiments.

### Preparation of anthraquinone derivatives

1,4-dihydroxyanthraquinone was dissolved in dimethylacetamide and pyridine at 0°C and dodecanoyl chloride added. The reaction mixture was stirred at 0°C to room temperature for 24 hrs. The light yellow precipitate was washed with hexane. Mitoxantrone (Quiral Quimica do Brasil S.A., Juiz de Fora, MG, Brazil) and its analog was solubilized in DMSO (Sigma, USA), never exceeding 0.1% (v/v) and diluted in 0.9% sterile saline.

### Induction of AAD and rhinovirus-induced exacerbation

Mice were intranasally sensitized with house dust mite extract (HDM; 50μg daily at day 0, 1 and 2) followed by intranasal challenges (5μg daily from day 14 to day 17) delivered in 50μl of 0.9% sterile saline. Non-sensitized mice received sterile saline only. Mice were euthanized 24hrs after the last allergen challenge.

Four groups of mice were studied: non-allergic (SALINE), HDM allergic and vehicle treated (VEHICLE, DMSO 0.1%), HDM allergic and mitoxantrone treated (MITOXANTRONE 1mg/kg as previously described[35]), HDM allergic and analog treated (ANALOG 1mg/kg) mice. Treatments (100mcl/mouse/day) were administered intraperitoneally from day 12 to day 17 during HDM challenges and after HDM sensitization. In another series of experiments mice were intranasally infected with minor group RV (RV1B) – 50µl containing 1x10^7^ virions – or UV-inactivated RV1B at day 18 which was 24hrs after the last HDM challenge to exacerbate preexisting AAD. Mice were euthanized 24hrs after the RV1B infection.

### AHR measurement

AHR was invasively assessed in separate groups of anesthetized mice by measurement of total lung resistance in response to increasing doses of methacholine as previously described[36]. Percentage increase over baseline (water) in response to nebulized methacholine was calculated. 

### Collection of bronchoalveolar lavage fluid

Twenty-four hours after the last HDM challenge, bronchoalveolar lavage (BAL) fluid was performed by cannulating the trachea and instillation of 800 µl of Hank's Buffered Salt Solution (HBSS) three times.

### Total and differential cell counts

BAL fluid was centrifuged at 800 x g for 10 min at 4°C and cell-free supernatant was collected. Cells were resuspended in 100µl of HBSS and total number of viable cells was determined by trypan blue exclusion in a Neubauer cell chamber. Cytospins were prepared and slides were stained with May-Grunwald-Giemsa. Differential cell counts were determined from a total of 200 cells per slide.

### Quantitative Real time PCR

Lower airway tissue from the left lung of each animal was separated by blunt dissection and stored in RNA later (Ambion, USA) at -80°C. Total RNA was extracted from the airways according to the manufacturer's instructions (mirVana m/miRNA isolation kit, Ambion, USA). The *primers* used were synthesized by Sigma (Table 1). qRT-PCRs reactions were performed using the SYBR® Green (Kappa Biosystems, USA) according to manufacturer's instructions. After amplification all samples were subjected to dissociation curve analysis in order to validate the absence of nonspecific products and primer dimers. RNA was normalized to expression levels of Hypoxanthine-guanine phosphoribosyl transferase (HPRT) and relative expression was calculated with the 2^-ΔΔCt^ method. 

**Table 1:** Primer sequences.

**Primer**	**Forward**	**Reverse**
CCL8	GGGTGCTGAAAAGCTACGAG	TTCCAGCTTTGGCTGTCTCT
CXCL10	CATTTTCTGCCTCATCCTGCTG	GGAGCCCTTTTAGACCTTTTTTGG
FOXP3	AGCAGTGTGGACCGTAGATGA	GGCAGGGATTGGAGCACTT
HIF1α	AGCTTCTGTTATGAGGCTCACC	TGACTTGATGTTCATCGTCCTC
HPRT	AGGCCAGACTTTGTTGGATTTGAA	CAACTTGCGCTCATCTTAGGCTTT
IFNα	GAACATCTTCACATCAAAGG	CAGAATGAGTCTAGGAGGGTTG
IFNβ	AAGAGTTACACTGCCTTTGCCATC	CACTGTCTGCTGGTGGAGTTCATC
IL-17A	ATCCCTCAAAGCTCAGCGTGTC	GGGTCTTCATTGCGGTGGAGAG
IL-17F	CTGTTGATGTTGGGACTTGCC	TCACAGTGTTATCCTCCAGG
IL-23p19	CCAGCAGCTCTCTCGGAATC	TCATATGTCCCGCTGGTGC
IL-6	TAGTCCTTCCTACCCCAATTTCC	TTGGTCCTTAGCCACTCCTTC
Muc5ac	GCAGTTGTGTCACCATCATCTGTG	GGGGCAGTCTTGACTAACCCTCTT
ROR-yT	CGCGGAGCAGACACACTTA	CCCTGGACCTCTGTTTTGGC
RV16/1B	AGTCCTCCGGCCCCTGAATG	AAAGTAGTYGGTCCCATCCGC
STAT3	AATATAGCCGATTCCTGCAAGAG	TGGCTTCTCAAGATACCTGCTC
STAT6	CTGGGAGTTCCTGGTCGGT	CTGTGGCAGAAAGTAGGGCAC
TNF-α	GTCTACTGAACTTCGGGGTGATCG	AGCCTTGTCCCTTGAAGAGAACCT
VEGF	CCAAGTGGTCCCAGGCTGCACC	GGTTAATCGGTCTTTCCGGTGAG

### Airway morphology analysis

The right lower lung lobe from each animal was fixed in 10% buffered formalin and the samples were subjected to routine histologic procedures and then stained with periodic acid-Schiff (PAS) to identify mucus glycoconjugates, Toluidine blue to identify mast cells, or Carbol’s chromotrope-hematoxylin to identify eosinophils. Cells were identified by morphological criteria and quantified by counting ten high-powered fields (HPF) in each slide.

### Measurement of cytokines

Peribronchial lymph nodes were excised, filtered and cultured in the presence of HDM (50μg/ml) for 6 days. Levels of IL-13, IL-5 and IFN-γ in supernatants were determined by ELISA (BD Biosciences Pharmingen, USA) according to the manufacturer's instructions. CD4+ T-cells were isolated from the draining lymph nodes using an Auto Macs Pro (Miltenyi Biotec, USA) according to the manufacturer’s instructions. Levels of IL-4 and IL-13 were measured concurrently by multiplex using the Novex platform (Invitrogen, USA) according to the manufacturer’s instructions before being quantified using a Bioplex (Biorad, USA) luminex system. Whole mouse lungs were homogenized with a Tissue Tearor (Biospec Products, USA) on ice in lysis buffer. 

### Flow cytometry

To prepare single-cell suspensions from whole lung and lymph nodes, tissues were gently mashed through 100µm cell strainers (BD Falcon). Red blood cells were removed using lysis buffer (4.15g ammonium chloride, 1g sodium hydrogen carbonate, 0.0185g EDTA in 500ml of dH_2_O). Cells were counted and the Fc receptor was blocked. Cell surface expression of CD4 (PE), CD8 (PerCP), TCRβ (FITC), CD3e (APC), CD19 (PerCP), CD11b (PerCP), CD11c (FITC), F4/80 (APC) and MHCII (PE) (all antibodies from Pharmingen, USA) was determined by flow cytometry analysis with a FACSCanto flow cytometer using commercially available Abs from BD Biosciences. Cells gated by forward- and side-scatter parameters were analyzed using FACSDiva software.

### p-AKT Western blot

Levels of p-AKT were determined by western blotting in whole cell protein lysates isolated from lung homogenates. Protein samples at 45 μg/lane underwent electrophoresis on a 10% SDS-polyacrylamide gel and were electroblotted onto PVDF. Membranes were blocked for 2h at room temperature in TBS containing 5% bovine serum albumin (Sigma), the membrane was incubated for 2h at room temperature with monoclonal anti p-AKT (1:300 in a TBST solution made up with 10ng/ml of B-Actin). After washing the membrane 3x for 5min in TBST the membrane was incubated with HEP-conjugated secondary antibody (1:5000 in TBST) for 1h at room temperature. The membrane was incubated with Luminata Cresecendo Western HRP Substrate (Millipore) and visualized on a Fujifilm LAS-4000 using Image reader LAS-4000.

### Determination of PIP_3_ activity

Levels of active phosphatidylinositol-(3,4,5)-trisphosphate (PIP_3_) were determined by ELISA (Echelon Biosciences, USA) according to the manufacturer’s instructions.

### Statistical analysis

Numerical data were analyzed for normal distribution employing the Kolmogorov-Smirnov test. Subsequently, the unpaired t test was used for parametric data or Mann Whitney test for nonparametric data. The significance level accepted for the tests was p<0.05. Data are expressed as mean ± standard error of the mean (SEM).

## Results

### Treatment with anthraquinones ameliorates hallmark features of AAD

**Figure 1:**
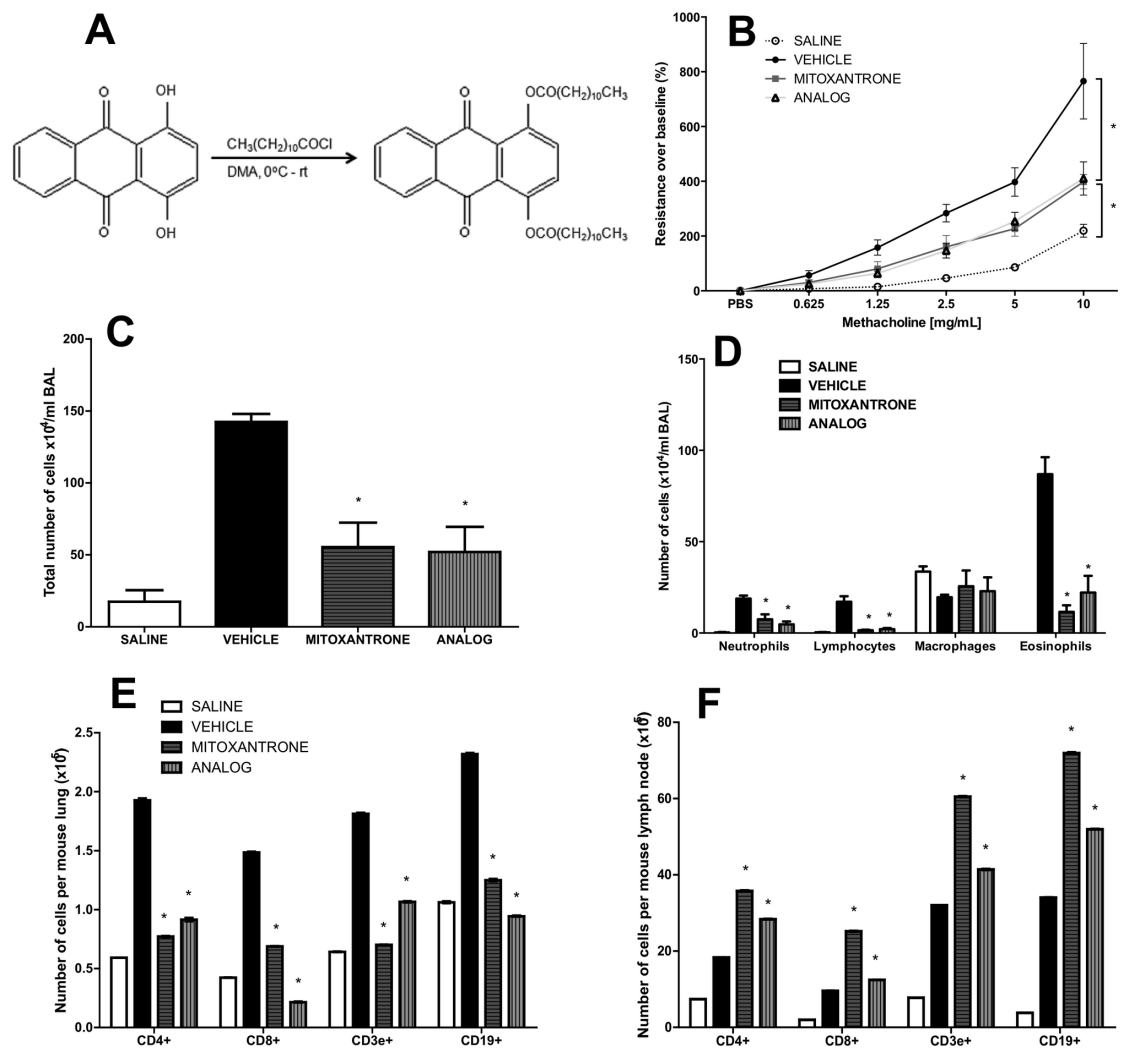
Anthraquinone derivative suppresses AHR and inflammation. (A) Scheme of chemical synthesis of the anthraquinone derivative *O*,*O*´-didodecanoyl-1,4-*dihydroxyanthraquinone*. (B) AHR, (C) total and (D) differential number of BALF cells, (E) T (CD4, CD8) and B (CD19) cell numbers in lung homogenates and (F) peribronchial lymph node cells in HDM sensitized and challenged mice treated with 1 mg/kg of mitoxantrone or analog. * p<0.05 when compared to vehicle. Data represent the mean±SEM of at least two independent experiments n=6.

**Figure 2:**
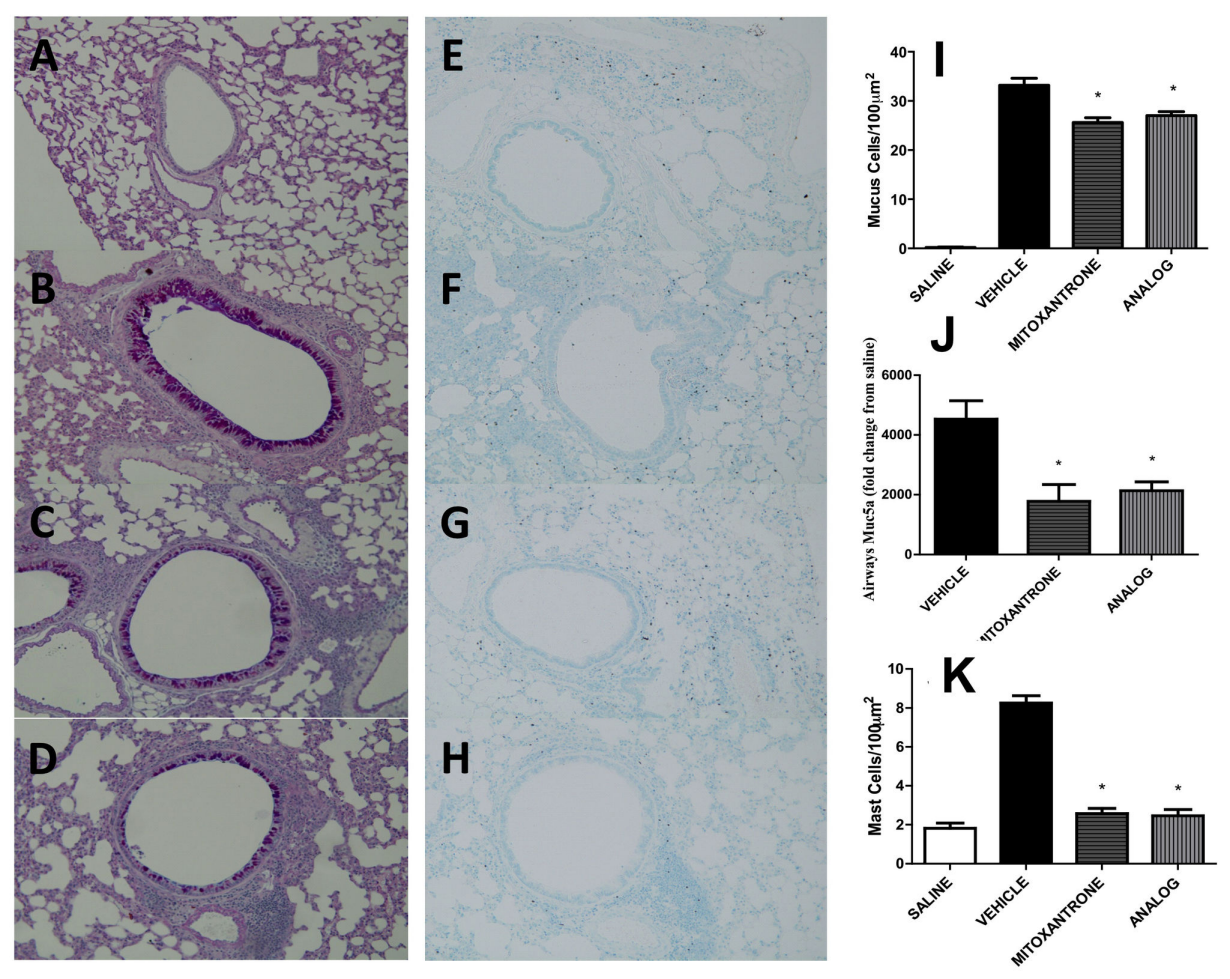
Mucus production and mast cell numbers in the airways are reduced by anthraquinone derivatives. Fixed airway sections stained with (A-D) Periodic acid-Schiff (PAS) staining or Toluidine blue (G-J) from non-allergic saline treated mice (A, E), vehicle (B, F), mitoxantrone (C, G) and analog (D, H) treated allergic mice; bar 50 μm. (I) Mucus cells counted in ten high-powered fields (100 µm^2^) of the PAS stained lungs; (J) RT-PCR with RNA isolated from lower airway tissue; data normalized to HPRT and the relative expression of Muc5ac calculated relative to saline; (K) Mast cell counts in ten high-powered fields (100 µm^2^) of the Toluidine blue stained lungs; * p<0.05 when compared to vehicle. Data represent the mean±SEM of at least two independent experiments n=6.

The ability of mitoxantrone to intercalate with the DNA through hydrogen binding was precluded by synthesizing a novel anthraquinone derivative, *O*,*O*´-didodecanoyl-1,4-*dihydroxyanthraquinone* (Figure 1A). Consequently, this analog did not exhibit any *in-vitro* cytotoxicity on transformed macrophage cell lines or cytotoxic effects *in-vivo* (data not shown). In order to investigate the anti-inflammatory properties of mitoxantrone and its analog on AAD, we sensitized and challenged BALB/c mice with HDM via the airway route which resulted in the development of AHR (Figure 1B) and increased cellularity in BAL fluid (Figure 1C) consisting of eosinophils, lymphocytes, and neutrophils (Figure 1D). Treatment with mitoxantrone or its non-cytotoxic analog significantly reduced AHR and airways inflammation (Figure 1C and D). To further investigate the effect of mitoxantrone and its analog on accumulation of lymphocyte subsets in the lungs and peribronchial lymph nodes (PBLN) FACS analysis was performed. Both mitoxantrone and its analog impaired recruitment of T cells (CD3+), CD4+ and CD8+ T helper cells, and CD19+ B cells into the lungs (Figure 1E) while those cells accumulated in the PBLN (Figure 1F). Mucus hypersecretion, Muc5ac expression, and mast cell influx were also significantly reduced upon mitoxantrone or analog treatment (Figure 2 A to K). 

### Anthraquinones impair expression of pro-inflammatory cytokines along with regulatory transcriptional factors in the lung

**Figure 3:**
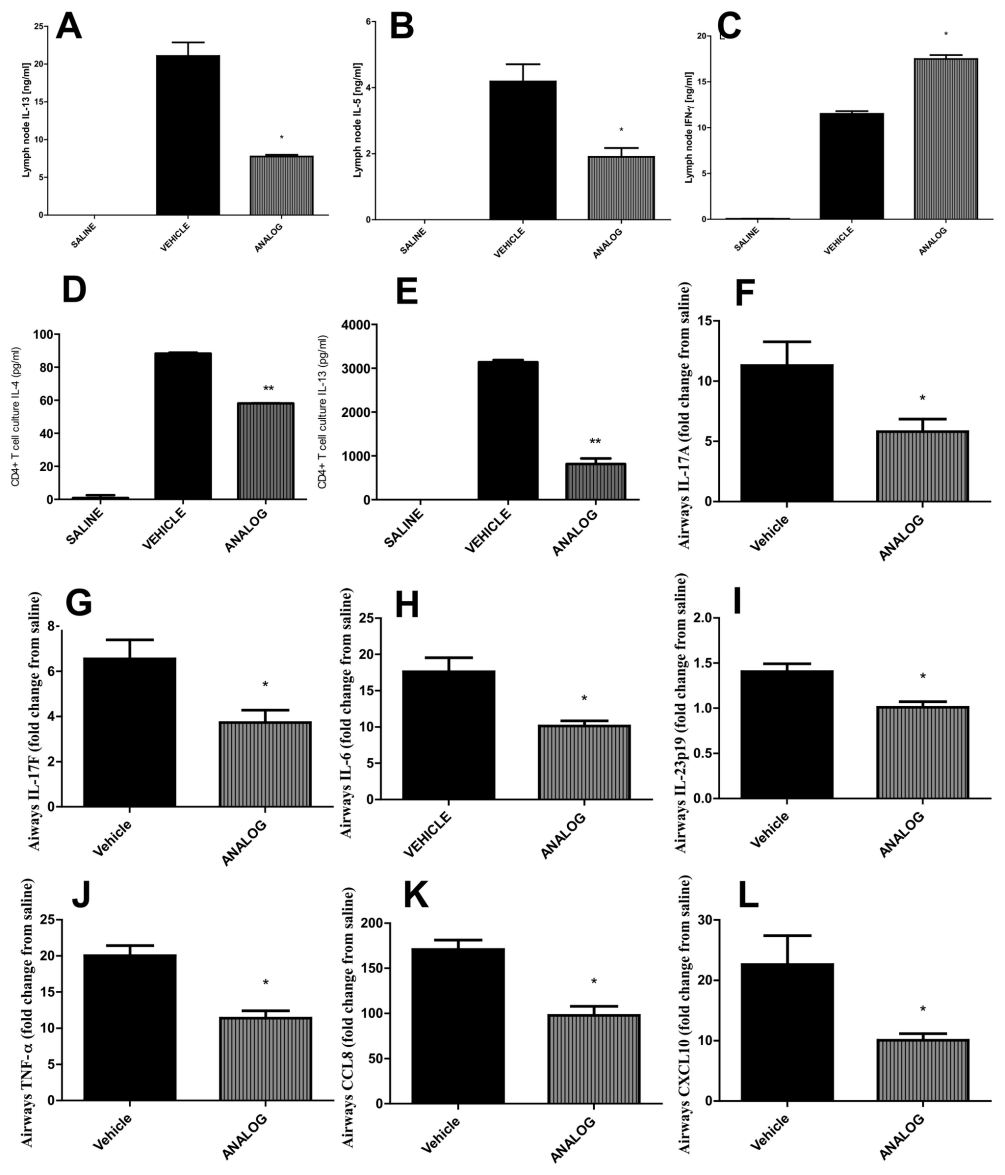
T_H_1/2/17 cytokine and chemokine expression upon treatment with anthraquinone derivative. (A) IL-13, (B) IL-5, and (C) IFN-λ release from peribronchial lymph node cells cultured in the presence of HDM (50µg/ml). (D) IL-4 and (E) IL-13 release form CD4+ T-cells cultured in the presence of HDM (50µg/ml). RT-PCRs with RNA isolated from lower airway tissue; data normalized to HPRT and the relative expression of (F) IL-17A, (G) IL-17F, (H) IL-6, (I) IL-23p19, (J) TNF-α, (K) CCL8 and (L) CXCL10 was calculated relative to saline. * p<0.05 when compared to vehicle. Data represent the mean±SEM of at least two independent experiments n=6.

**Figure 4:**
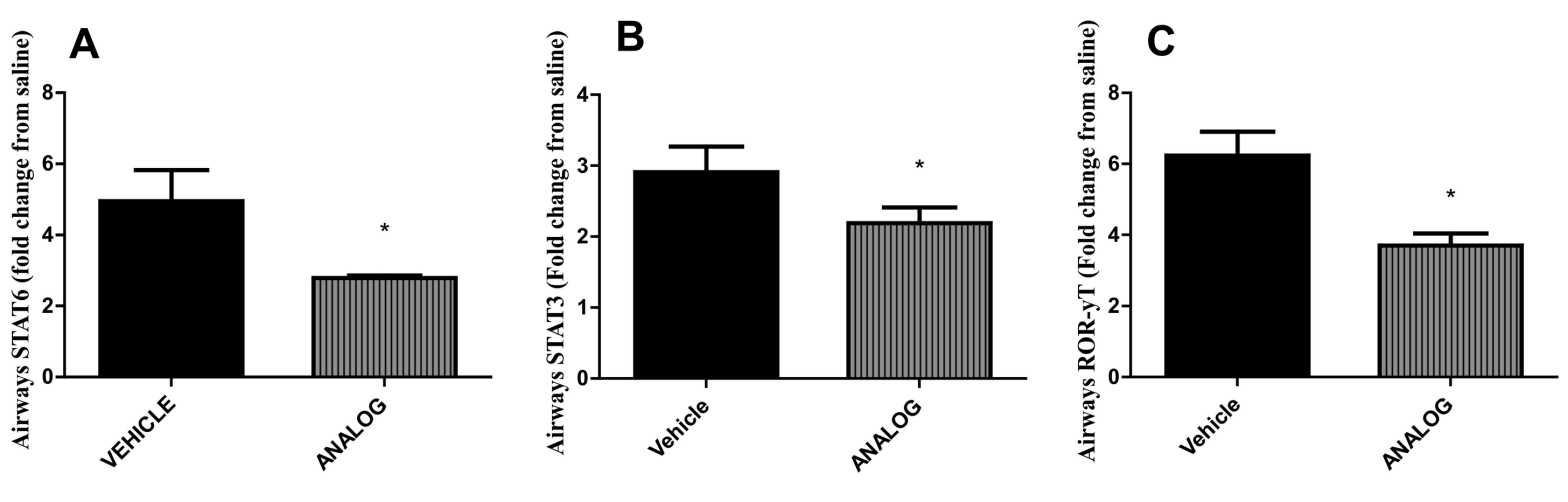
Expression of transcription factors after anthraquinone derivative. BALB/c mice were sensitized and challenged with HDM intranasally and treated with 1 mg/kg of analog. RT-PCRs with RNA isolated from lower airway tissue; data normalized to HPRT and the relative expression of (A) STAT6, (B) STAT3, (C) ROR-γT and (D) FOXP3 was calculated relative to expression in saline. * p<0.05 when compared to vehicle. Data represent the mean±SEM of at least two different experiments n=6.

IL-13 and IL-5 release from *in-vitro* HDM-stimulated PBLN cells was significantly impaired in allergic mice treated with the antraquinone analog while IFN-γ release was increased (Figure 3 A to C). HDM-stimulated CD4+ T cells isolated from mice treated with the antraquinone analog released lower levels of the archetypal T_H_2 cytokines IL-4 and IL-13 (Figure 3 D to E). Expression of cytokines promoting T_H_17 immunity such as IL-17F, IL-17A, IL-6, and IL-23p19 were also reduced (Figure 3 F to I) but we were not able to detect IL-17 protein in CD4+ T cell or PBLN supernatants, or lung homogenates. Other pro-inflammatory factors such as TNF-α, CCL8 and CXCL10 were also reduced in analog treated allergic mice (Figure 3J and K). Same results were observed with mitoxantrone-treated mice (data not shown). The reduction in T_H_2 and T17 cytokine expression upon analog treatment was associated with impaired expression of STAT6, STAT3, and ROR-γT (Figure 4A to C). 

### p-AKT phosphorylation, HIF-1α and VEGF expression are limited by anthraquinone treatment

**Figure 5:**
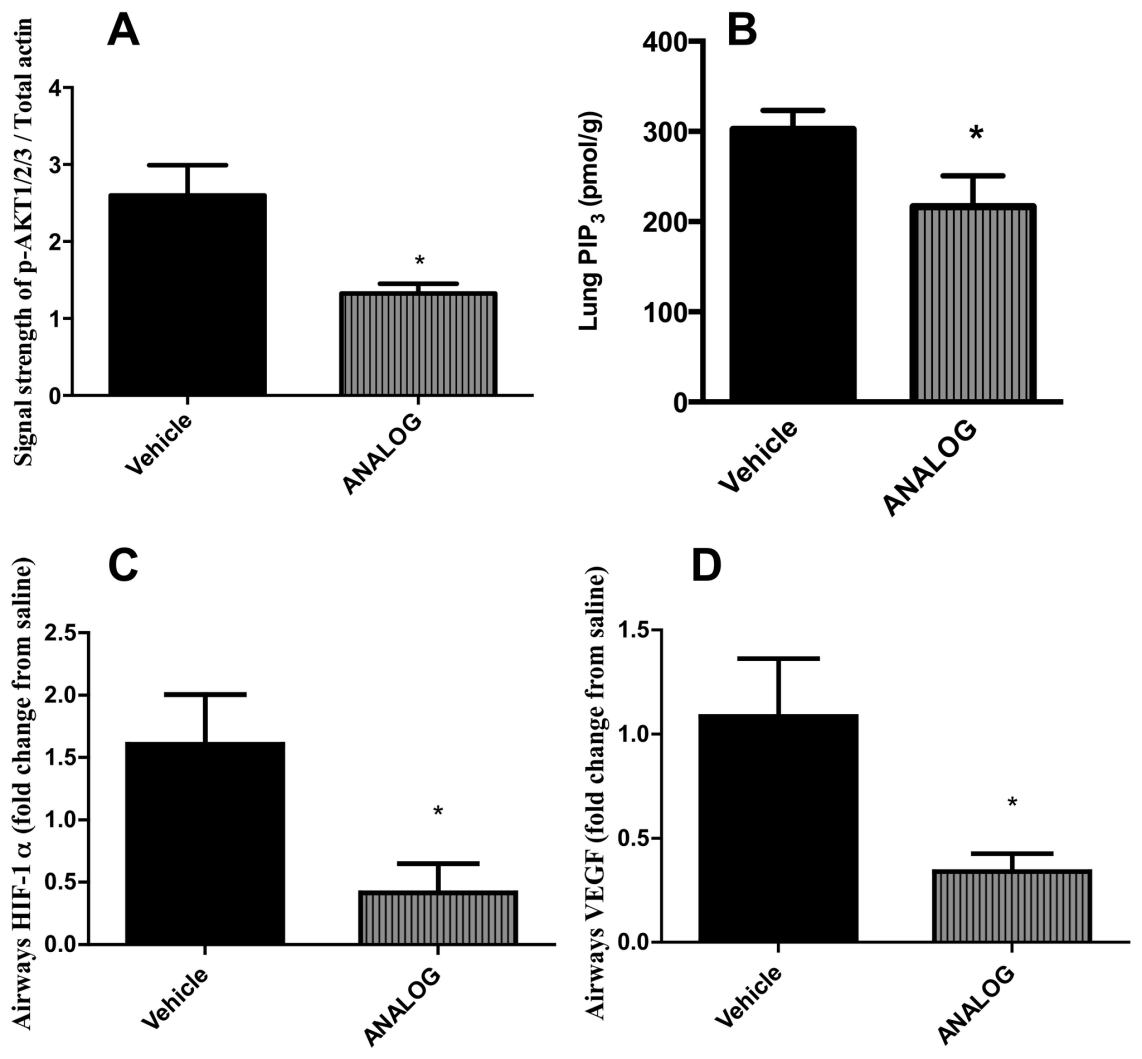
Phosporylated AKT, PIP_3_, HIF-1α, and VEGF expression after anthraquinone derivative treatment. (A) Protein lung lysates were isolated from allergic mice and levels of p-AKT1/2/3 were determined by western blotting. Signal strength of p-AKT when compared to total actin level. (B) PIP_3_ activity in lung lysates. (C-D) RT-PCRs with RNA isolated from lower airway tissue; data normalized to HPRT and the relative expression of (C) HIF-1α and (D) VEGF was calculated to saline expression levels. * p<0.05 when compared to vehicle. Data represent the mean±SEM of at least two independent experiments n=6.

In accordance with *in vitro* observations [26] anthraquinone treatment of allergic mice reduced HIF-1α and VEGF expression along with reduced levels of p-AKT and active PIP_3_ in the lungs (Figure 5A to D). 

### RV-induced exacerbation of AAD and RV replication are reduced by anthraquinone treatment

**Figure 6:**
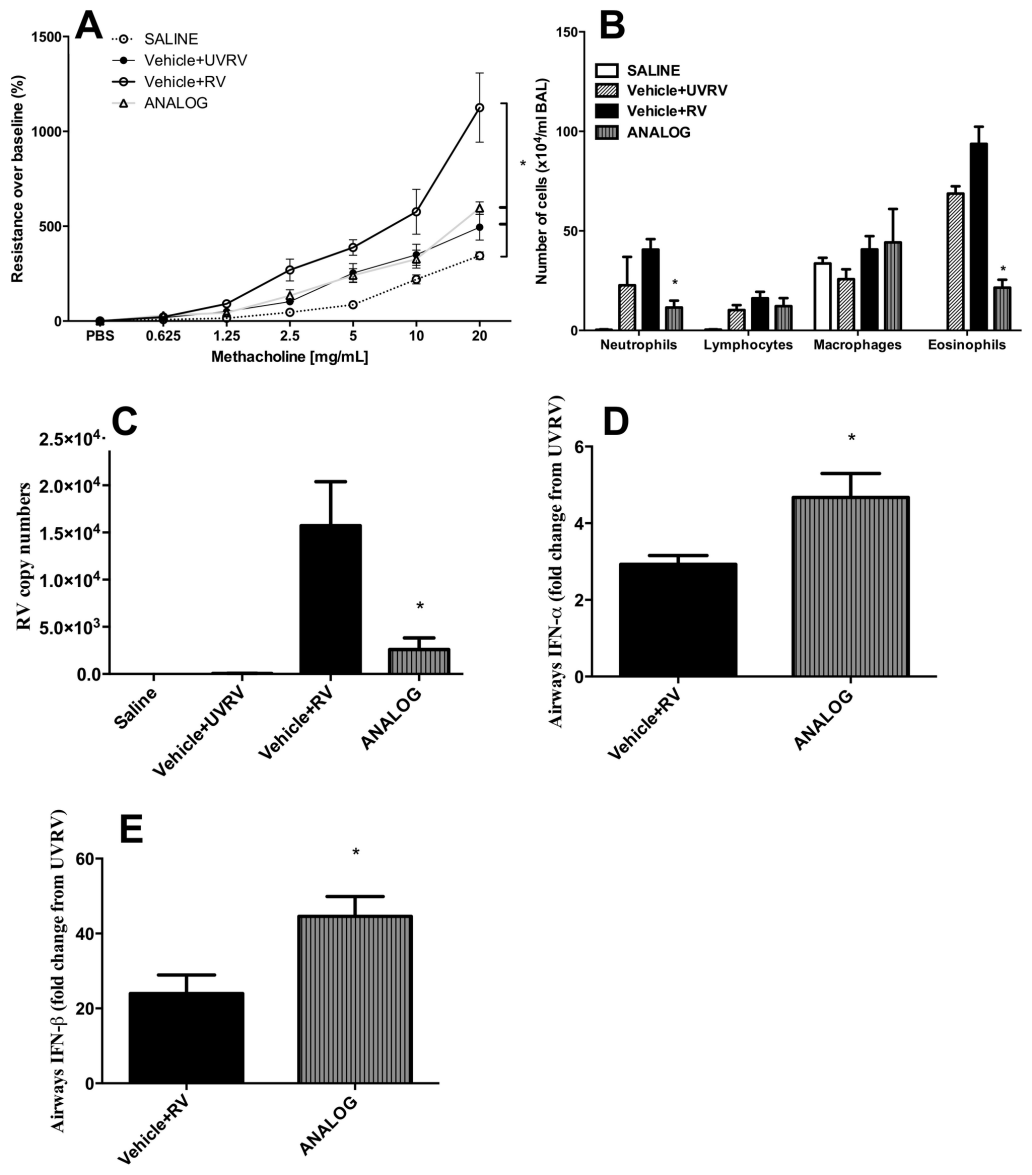
Rhinovirus-induced exacerbation of AAD is ameliorated by anthraquinone derivative treatment. 24hrs after the last HDM challenge, allergic mice were infected with 50µl of RV1B (RV) or UV-inactivated RV1B (UVRV). (A) 24hrs after RV1B infection AHR was determined. (B) Differential number of cells in the BALF. RT-PCRs with RNA isolated from lower airway tissue; data normalized to HPRT, (C) the absolute copy numbers of RV, and the relative expression of (D) IFN-α and (E) IFN-β was calculated relative UVRV expression levels. * p<0.05 when compared to vehicle+RV. Data represent the mean±SEM of at least two independent experiments n=6.

AHR is further exacerbated by RV1B infection of allergic mice as compared to mice exposed to UV-inactivated RV1B (Figure 6A). Notably, treatment with one dose of analog 24hrs before RV exposure resulted in marked attenuation of AHR to levels that are comparable to allergic mice exposed to UV-inactivated RV1B (Figure 6A). This was associated with inhibition of RV-induced exacerbation of eosinophilic and neutrophilic airways inflammation (Figure 6B). Interestingly anthraquinone treatment also impaired RV1B replication (Figure 6C) and increased expression of innate antiviral type 1 IFNs (Figure 6D and E). 

## Discussion

In the present work, treatment with *O*,*O*´-didodecanoyl-1,4-*dihydroxyanthraquinone*, a new non-cytotoxic anthraquinone derivative and analog of mitoxantrone, reduced the characteristic hallmark features of AAD including AHR, airways inflammation with eosinophils, neutrophils and mast cells, T-cell recruitment into the lung, expression of T_H_2 and T_H_17 transcriptional factors, release of T_H_2 cytokines, mucus hypersecretion, and RV-induced exacerbation. Moreover, anthraquinone treatment boosted antiviral IFN responses and limited RV replication. 

There is a need for improved therapies for patient with severe asthma as it accounts for 5% to 10% of cases and causes 50% of total asthma-related health costs [35]. For instance, humanized antibodies that ameliorate T_H_2 effector pathways by blocking circulating IgE (omalizumab) or IL-5 (mepolizumab) reduced exacerbations and need for other medication and improved asthma control [37–41]. Anti-IL-13 treatment (lebrikizumab) was associated with improved lung functions [42]. 

Severe asthma is also associated with increased IL-17A production [43] and studies in experimental models suggest a critical role for complement-mediated regulation of the IL-23-TH17 axis by enhancing IL-13-driven responses [44]. IL-17-expressing cells may promote neutrophilic inflammation, which is related to low lung function, worse asthma control, and increased exacerbations [43,45,46]. Children with severe asthma also displayed reduced levels of IFN-γ in their BALF, which –among other markers- best characterized severe versus moderate asthma [47]. Thus, severe asthma is not simply a T_H_2-mediated disease but may involve aberrant activation of T_H_17 cells [48] and accumulation of neutrophils in the airways [49]. Notably, our experimental studies could suggest that anthraquinones affects the IL-23-TH17 axis because we found reduced expression of IL-17F, IL-17A, IL-6, and IL-23p19. However, we could not detect IL-17 protein in lung samples or PBLN or CD4+ cell supernatant precluding us to conclusively investigate the effect of anthraquinonones on this pathway. 

RV-induced exacerbation of asthma is common and responds only partially to steroid treatment resulting in hospitalizations and contributing to asthma mortality. We have observed in our experimental HDM-mediated AAD model of RV-induced exacerbation that the development of AHR is only partially inhibited by systemic dexamethasone treatment and neutrophilic inflammation remains unaffected while RV-induced eosinophil influx is greatly reduced (data not shown). We show here that steroid-resistant, RV-induced AHR and neutrophilic inflammation are abolished by treatment with one dose of mitoxantrone analog before RV exposure. This effect may be explained by a boost in IFN production with subsequent inhibition of RV replication (Figure 6) as a consequence of therapeutically modulating T_H_ cell effector pathways. This conclusion is supported by studies in asthmatics that showed an inverse relationship between RV replication and IFN production [50]. Alternatively the deficiency in IFN production upon RV infection -which is observed in some asthmatics [15,16]- may promote aberrant T helper cell activation [51]. 

Kinases such as p38 mitogen-activated protein kinase, Jun kinases and PI3K play a key role in regulating inflammatory gene expression in asthmatics [52]. PI3K activation also results in reduced steroid sensitivity through phosphorylation of AKT and subsequent reduced histone deacetylase 2 activity [53]. PI3K inhibitors against isoforms attenuate antigen-induced airway inflammation in murine models [29,54] and might reverse steroid resistance in severe asthmatics [52]. Unselective PI3K inhibitors however are likely to be toxic [55]. 

Mitoxantrone has recently been shown to block PI3K signaling through dephosphorylation of AKT *in-vitro* resulting in reduced HIF-1α and VEGF expression [26]. We have shown here that a non-cytotoxic anthraquinone derivative attenuates the PI3K signaling pathway *in-vivo* despite its inability to disrupt DNA synthesis and repair due to removal of the hydrogen molecules during chemical synthesis (Figure 1A). Interestingly, Dang et al have recently demonstrated that HIF-1α plays an important role in the alternate induction of T_H_17 cells [56]. Furthermore inhibition of HIF-1α attenuated OVA-induced AHR and inflammation via VEGF suppression in bronchial epithelium [57], which is a critical effector molecule in Th2 driven allergic disease in the lung [58,59]. 

Recently Emodin and Citreorosein, two naturally occurring anthraquinone derivatives, have been shown to suppress IgE-mediated anaphylactic reaction, mast cell activation, and leukotriene generation [60,61]. This was associated with blockage of antigen-triggered phosphorylation of Syk, a receptor-proximal tyrosine kinase targeted by anthraquinones, which regulates the PI3K pathway through the adaptor protein NTAL [62] and plays a key role in NF-κB-mediated expression of COX-2 and pro-inflammatory cytokines. 

Interestingly, Syk is also phosphorylated upon RV binding to its cell surface receptor and regulates clathrin-mediated RV endocytosis through the activation of the PI3K/AKT signaling pathway [63]. Our results highlight the *in-vivo* relevance of this pathway by demonstrating limited RV replication, AHR and airways inflammation in allergic mice treated with anthraquinone derivatives. 

**Figure 7:**
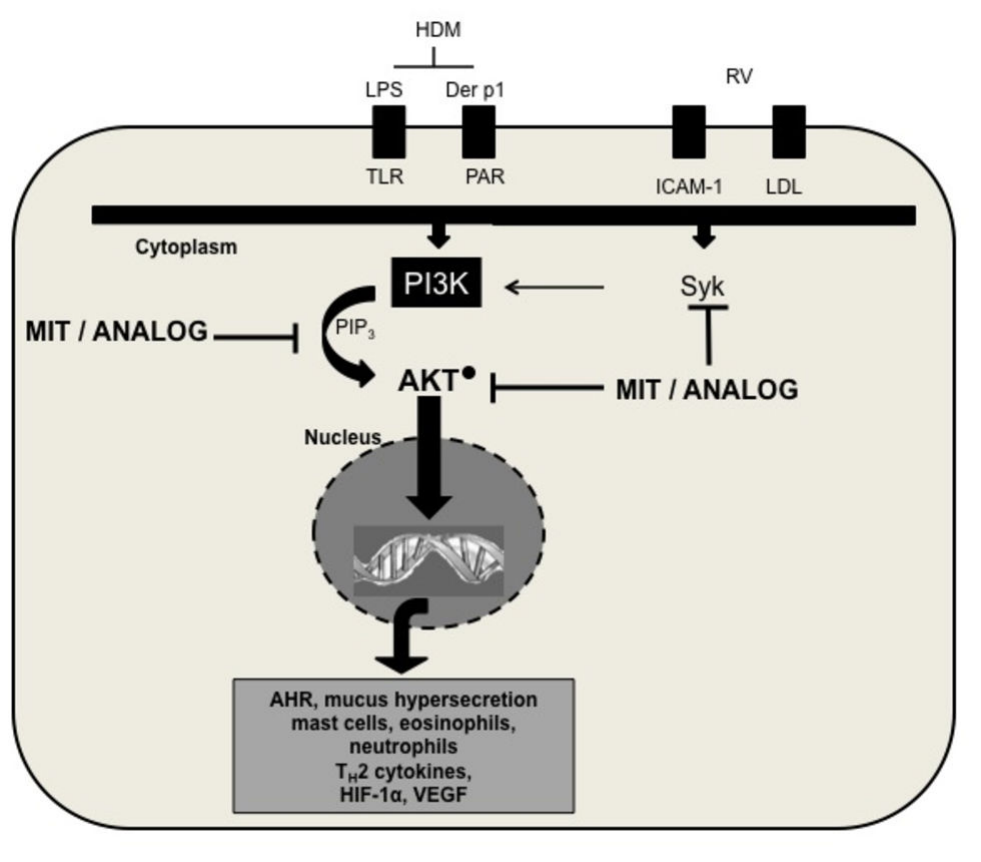
Proposed mechanism of *O*,*O*´-didodecanoyl-1,4-*dihydroxyanthraquinone* action.

The PI3K/AKT signaling pathway is also activated by HDM allergen Der p1 binding to protease-activated receptors [64] and LPS (e.g. in crude HDM extract) sensing by TLR4 [65]. Thus employing non-cytotoxic anthraquinone derivatives may be of therapeutic benefit for the treatment of both allergen- and RV-triggered severe asthma (Figure 7).
